# A computational framework to improve cross-platform implementation of transcriptomics signatures

**DOI:** 10.1016/j.ebiom.2024.105204

**Published:** 2024-06-19

**Authors:** Louis Kreitmann, Giselle D'Souza, Luca Miglietta, Ortensia Vito, Heather R. Jackson, Dominic Habgood-Coote, Michael Levin, Alison Holmes, Myrsini Kaforou, Jesus Rodriguez-Manzano

**Affiliations:** a Section of Adult Infectious Disease, Faculty of Medicine, Imperial College London, London, W12 0NN, United Kingdom; b Centre for Antimicrobial Optimisation, Department of Infectious Disease, Faculty of Medicine, Imperial College London, London, W12 0NN, United Kingdom; c Section of Paediatric Infectious Disease, Faculty of Medicine, Imperial College London, London, W2 1NY, United Kingdom; d Centre for Paediatrics and Child Health, Imperial College London, London, W2 1NY, United Kingdom

**Keywords:** Host-response, Transcriptomic signatures, Diagnostics, Molecular test, RNA sequencing, Nucleic acid amplification techniques, PCR-based technologies, Multiplex PCR

## Abstract

The emergence of next-generation sequencing technologies and computational advances have expanded our understanding of gene expression regulation (i.e., the transcriptome). This has also led to an increased interest in using transcriptomic biomarkers to improve disease diagnosis and stratification, to assess prognosis and predict the response to treatment. Significant progress in identifying transcriptomic signatures for various clinical needs has been made, with large discovery studies accounting for challenges such as patient variability, unwanted batch effects, and data complexities; however, obstacles related to the technical aspects of cross-platform implementation still hinder the successful integration of transcriptomic technologies into standard diagnostic workflows. In this article, we discuss the challenges associated with integrating transcriptomic signatures derived using high-throughput technologies (such as RNA-sequencing) into clinical diagnostic tools using nucleic acid amplification (NAA) techniques. The novelty of the proposed approach lies in our aim to embed constraints related to cross-platform implementation in the process of signature discovery. These constraints could include technical limitations of amplification platform and chemistry, the maximal number of targets imposed by the chosen multiplexing strategy, and the genomic context of identified RNA biomarkers. Finally, we propose to build a computational framework that would integrate these constraints in combination with existing statistical and machine learning models used for signature identification. We envision that this could accelerate the integration of RNA signatures discovered by high-throughput technologies into NAA-based approaches suitable for clinical applications.

## Introduction

Gene expression microarrays and whole-transcriptome sequencing, also known as RNA-Sequencing (RNA-Seq), are high-throughput technologies used to quantify the expression of tens of thousands of RNA molecules (the transcriptome) in biological samples, simultaneously and in a hypothesis-free manner. They provide valuable insights into the expression of an organism's genes or transcripts in different cells and tissues at different time points, and have been instrumental in understanding cellular differentiation and gene expression regulation.^1^ Transcriptomics has also seen a broad range of medical research applications and holds great promise to address unmet clinical challenges in a precision medicine approach. Clinical applications of transcriptomics are based on the premise that the granularity of gene expression analysis can be leveraged to obtain a level of information on disease classification, stratification, prognosis or expected response to treatment that is beyond the reach of existing tools.^2^

The development of host RNA-based diagnostic tests starts with the data-driven discovery of a gene signature to address a clinical need. To ensure the robustness and reproducibility of the signature, the discovery process entails enrolling patient cohorts with clearly defined phenotypes, carefully considering biological variability stemming from factors like genetics, disease heterogeneity and microbial interactions. Subsequently, it involves the generation and curation of gene expression data via high-throughput technologies (i.e., the **discovery platform**). Bioinformatic analyses are then applied to pinpoint sparse and precise biomarker sets, requiring advanced analytical techniques to discern relevant biomarkers while mitigating the risks of false discoveries and overfitting. In addition, when multiple patient cohorts or publicly available datasets are being used for discovery, elimination of systematic non-biological differences (called ‘batch effect’) between datasets is key.^3^^,^^4^

**Fig. 1:**
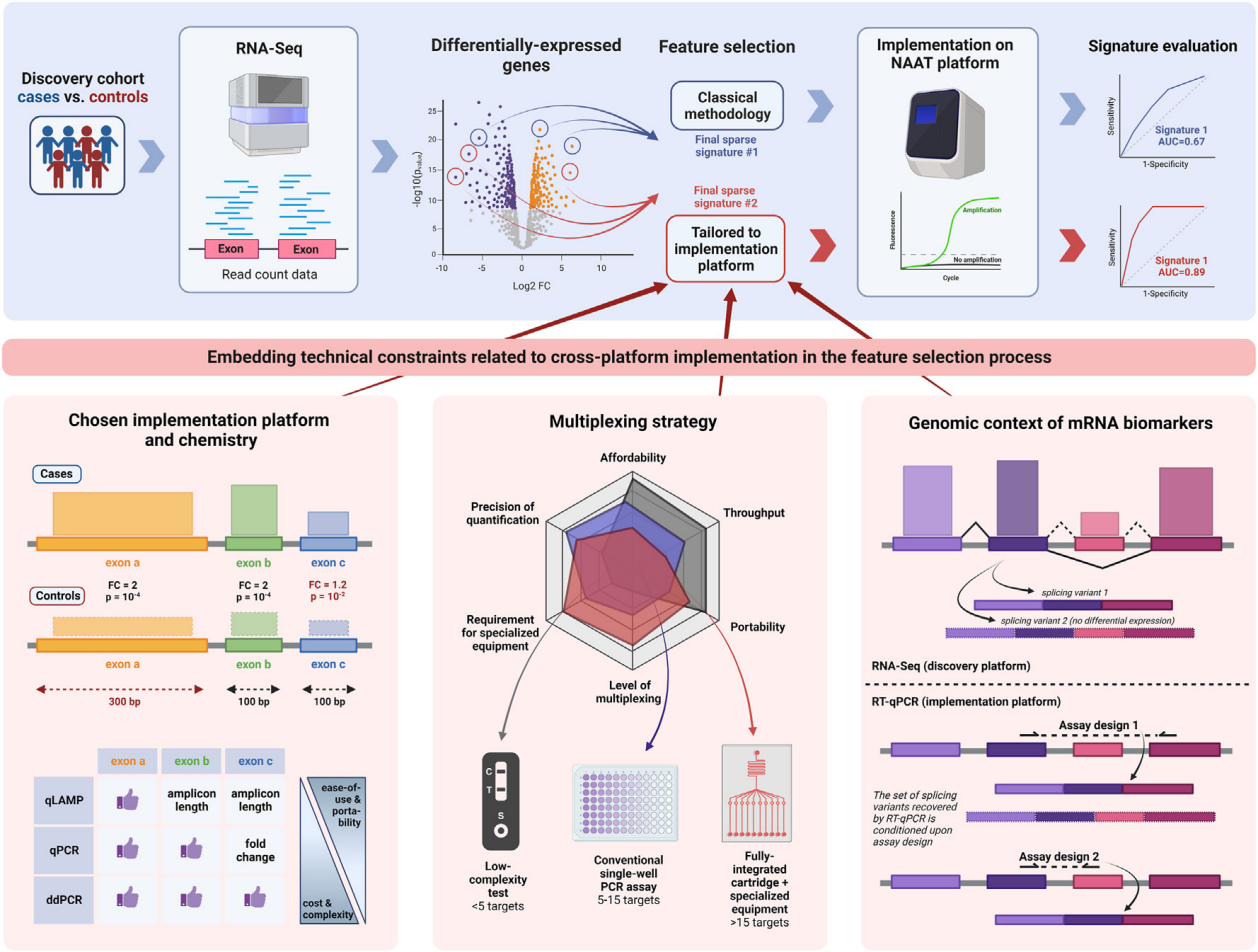
**Embedding constraints related to cross-platform implementation in the feature selection process to improve the translation of transcriptomics signatures into diagnostic tools**. In this figure, we depict classical methodologies for gene signature discovery and implementation (blue background), and propose novel strategies to ensure that signatures retain optimal classification accuracy after cross-platform transfer (red background). Classically, the down-selection of a large set of differentially expressed genes in a final sparse signature is mainly based on statistical criteria and machine learning methods (top blue panel). Here, we propose to enhance this feature selection process by designing algorithms that would further incorporate constraints associated with the target implementation platform and chemistry. In the bottom left panel, we highlight how three exons with different characteristics (differential expression in cases vs. controls and length) may be suited for quantification using real-time LAMP (qLAMP), reverse transcription real-time PCR (RT-qPCR) and digital droplet PCR (ddPCR). In the bottom middle panel, we present how different multiplexing strategies might impact the total number of transcripts included in the final sparse signature. In the bottom right panel, we show that different assay designs (i.e., the location of primers on the genomic region of interest) might impact the way splicing variants are quantified on the discovery and implementation platform (RNA-Seq vs. RT-qPCR). Taken together, we suggested embedding these criteria upstream of the discovery process to ensure successful implementation of transcriptomics signatures into validated diagnostic tools. NAAT: nucleic acid amplification technique, AUC: area under the receiver operating characteristic curve, FC: fold-change, qLAMP: quantitative loop-mediated isothermal amplification, RT-qPCR: reverse transcription real-time polymerase chain reaction (PCR), ddPCR: digital droplet PCR. Figure created with BioRender.

However, it is important to note that the successful implementation of transcriptomic signatures into diagnostic tests on a platform suitable for clinical use, referred to as the **implementation platform**, is also contingent upon a critical step of **cross-platform transfer** (see Fig. 1). Integrating considerations of cross-platform consistency alongside other crucial factors ensures a comprehensive approach to biomarker discovery and facilitates their translation into clinical practice. Despite considerable technological advancements and decades of research ultimately aimed at applying this approach to patient management, there remain notable challenges on the path from signature discovery to test implementation. Attesting to this is the discrepancy between the abundance of published signatures and the low number of validated and commercially available diagnostic tests based on host RNA molecules.^5^^,^^6^ There are various reasons contributing to this gap, including cost, accessibility, technical challenges, regulatory and standardisation hurdles, along with reduced performance in external validation studies in some cases. The reduced performance observed in validation studies can be attributed to biological complexity and inter-individual variability due to genetic differences or environmental factors, as well as intra-individual variability and temporal differences in gene expression influenced by metabolic changes, drugs and other diseases. However, even when these factors are carefully accounted for, variable results have been obtained when attempting to implement the results of transcriptomic studies for clinical use. More precisely, we and others have observed and reported a decline in the diagnostic or predictive performance of RNA-based signatures when they are evaluated on their implementation platform, in comparison to their initial discovery platform.^7^^,^^8^ Importantly, this might not have been reported nor emphasized in the literature as studies where biomarkers perform poorly at the validation/implementation stage generally do not reach publication stage. Additionally, we have also repeatedly observed that identical samples from the same patients could yield different RNA quantification results (and consequently a reduced performance of any RNA-based diagnostic metric) when processed on different platforms.

Motivated by these observations, in this article we are advancing the idea that this ‘failure of implementation’ or ‘lack of transferability’ may be partly due to **a decoupling between the steps of signature discovery and cross-platform transfer.** While the primary focus of past studies has been on improving processes, methods and technologies to discover signatures with optimal classification accuracy, there has been comparatively limited research on the factors underlying the successful implementation of these signatures in conventional techniques like the polymerase chain reaction (PCR), thus impeding the translation of scientific discoveries into clinical solutions. To illustrate our point, we will briefly describe current practices in transcriptomic biomarker discovery research; we will highlight limitations in the way existing strategies have failed to embed characteristics and constraints of implementation platforms and chemistries in the process of signature discovery; and finally, we will suggest avenues of future research.

## Current practices for discovery of gene signatures

In brief, bulk RNA-Seq can accurately measure relative gene, exon, splice junction and transcript abundance, thereby generating highly dimensional datasets in a hypothesis-free manner. Following data quality control and pre-processing, genes are filtered for those with low expression and those with little or no association with comparator groups of interest (i.e., **disease classes** such as cases vs. controls, or patients with a favourable vs. poor prognosis).

Subsequently, **feature selection** is carried out to refine the number of messenger RNA (mRNA) biomarkers and obtain a final signature displaying an optimal trade-off between sparsity and classification accuracy. Importantly, this step of **feature selection** is mostly based on concepts and methodologies from the fields of statistics and machine learning, including fold-change (FC), p-value, or mean expression value. There is a plethora of feature selection and classification methods, and the choice of what method to apply is based on the experimental design and specific requirements associated with the clinical question being addressed.^9^, ^10^, ^11^ It is important to emphasize that the feature selection process typically does not account for the constraints associated with the phase of cross-platform transfer, such as technical limitations inherent to the chosen implementation platform and chemistry, multiplexing strategy, and the genomic context of identified mRNA biomarkers. The final steps include prediction model implementation and performance evaluation, usually carried out using independent cohorts.

Validation of the diagnostic performance of RNA-based diagnostic tests using an independent validation cohort but the same RNA quantification platform that was used for discovery is important to verify that the signature is not ‘cohort-specific’, which could arise if the discovery cohort is not representative of the population to which the test is applied. It is important to note, however, that this step is necessary but not sufficient for successful clinical translation: even after validation has been obtained on an independent cohort (and the same platform), successful implementation in a diagnostic tool requires careful consideration of the platform chosen for clinical use.

## Technical limitations of implementation platform and chemistry

High-throughput technologies, which play an instrumental role in the discovery of RNA signatures, are not appropriate for clinical use because of their high cost, slow turn-around time and the requirement for specialized equipment and trained personnel. To inform prompt clinical decisions, the measurement of gene expression must be conducted on more accessible platforms, often based on targeted nucleic acid amplification tests (NAATs).^12^ Among these, real-time PCR (qPCR), preceded by a step of reverse transcription (RT-qPCR), is the most standard tool due to its large dynamic range of quantification, high sensitivity, specificity, and reproducibility. Novel NAATs sharing the common feature of being isothermal (i.e., capable of amplifying DNA at a constant temperature), such as loop-mediated isothermal amplification (LAMP), have emerged in the last decades as alternatives to PCR, particularly suited for point-of-care applications.^13^ More recently, digital PCR (dPCR), a technology based on the partitioning of input samples into thousands of micro- or nano-compartments or droplets, has been evaluated to reach absolute quantification of signature transcripts.^14^ Finally, strategies based on the use of machine learning^15^ and thermodynamic models^16^ to enhance multiplexing and improve quantification in PCR have also been developed, and molecular techniques based on electrochemical sensing have been investigated to improve the portability of NAATs at the point of care.^17^

Independently of the chosen implementation chemistry or platform, accurate quantification of transcript abundance using NAATs (which is critical for successful cross-platform transfer) relies on the amplification of a region of the target mRNA (the amplicon), delineated by sequence-specific primers. In theory, any nucleic acid sequence can be targeted as a site for primer binding in PCR or LAMP. However, the successful implementation of a given NAAT depends on meeting a range of biochemical and thermodynamic criteria, such as primer melting temperature, amplicon length, GC content, specificity of primer binding on the region of interest, or avoidance of primer-dimers (especially in the case of single-tube multiplex PCR assays, see section below). Importantly, these constraints may drastically limit the potential for certain transcripts to be included in a NAAT-based diagnostic test. As an example, it might be challenging to design reliable primers for an exon displaying highly significant differential expression across classes, but unusually high GC content. Thus, incorporating biochemical and thermodynamic criteria that can impact **molecular assay design**, in addition to purely statistical criteria, into the feature selection process leading to the final sparse signature, could ensure that classification performance is maintained on the implementation platform. Importantly, these constraints may differ across **implementation chemistries**. For instance, the minimal amplicon length necessary for successful implementation on a LAMP-based platform might be longer than on a PCR-based platform, since a typical LAMP assay usually relies on a total of six primers targeting eight genomic regions per amplicon and spanning across 200–250 base pairs.^18^ Additionally, LAMP, while advantageous in terms of simplicity, speed, and cost-effectiveness, particularly for rapid and sensitive detection of nucleic acids in resource-limited settings, presents challenges when combining with probes or performing melting curve analysis, which are commonly used in PCR assays for enhanced specificity and multiplexing capabilities.^19^

Considering the chosen **implementation platform** upstream in the discovery process is also of paramount importance. For instance, one of the main constraints imposed by the choice of a given implementation platform is its dynamic range of quantification, defined as the ability of a platform to measure a range of input DNA/RNA concentrations with sufficient precision (i.e., low technical variability). Digital PCR will classically provide higher precision of quantification than qPCR, even when used on identical nucleic acid targets and molecular assays. However, the dynamic range of quantification in dPCR might be limited by the number of partitions, which varies across commercial solutions. Thus, in the case of an exon having a greater than a 2-fold difference in expression levels between comparator disease groups but an overall high mean expression level, qPCR would serve as an optimal implementation platform, whereas dPCR would be more suited for an exon showing a 1.5-fold difference of expression levels across classes but having an overall low mean expression level.

In another example, the use of a PCR-based platform designed for lab-based versus point-of-care applications may dictate the complexity of upstream sample preparation, size of the signature (e.g., number of markers), and abundance of targets. Lab-based PCR platforms, with their higher throughput capacity and advanced features, may be better suited for comprehensive signatures and non-optimal sample types (e.g., low RNA quality or quantity, degraded RNA, or samples from tissues or cell types known for their complex composition or difficulty in RNA extraction). Conversely, point-of-care PCR platforms, which prioritize portability, cost, simplicity, and rapid results, making them ideal for decentralized healthcare settings, will often lack precise quantification capabilities and are limited to fewer reactions compared to their lab-based counterparts, which can potentially impact the accuracy and reliability of results.

## Multiplex strategy

The process of down-selection from a large set of differentially expressed genes to a sparse signature aims to find an optimal balance between parsimony and loss information, as well as to reduce the risk of over-fitting. In most disease contexts—especially when the signature is used to differentiate between two disease classes—a limited number of RNA biomarkers is sufficient to attain high classification accuracy.^11^^,^^20^ As an example, a fingerstick blood test based on mRNA expression of 3 genes has recently shown to have optimal performance (area under the ROC curve (AUC) of 0.94, 95% CI 0.91–0.97) for the diagnosis of tuberculosis in adults,^21^ but its performance was less optimal in a paediatric cohort.^22^ However, to address more complex clinical questions, for example differentiating between multiple classes using a single test, a higher number of transcripts need to be measured simultaneously.^23^^,^^24^

The simultaneous detection and precise quantification of several RNA biomarkers on a diagnostic platform remains challenging. Most commercially available technologies use microfluidic approaches, where initial samples are partitioned into sub-compartments where amplification and quantification take place in parallel single–plex reactions. An important intrinsic limitation of this *spatial multiplexing* approach is that a higher number of wells necessitates higher amounts of initial template for the reaction to remain above the lower limit of quantification, which can be problematic for transcripts with a low average expression level. Further, these technologies are not cost-efficient and have limited throughput (i.e., one sample per cartridge, processed by one end-user at a time for each sample).

*Single-well* multiplex NAATs, which combine multiple primer sets in one assay and differentiate amplified targets using post-PCR processes (e.g., use of fluorophores, melting curve analysis), can overcome some of these obstacles, but are more susceptible to non-specific interactions between primers (primer-dimers) and between primers and templates (other than their specific target), which can introduce a quantification bias. Practically, this means that two exons identified as appropriate mRNA biomarkers in RNA-Seq might impose significant challenges for assay designers aiming to combine them in a single-well multiplex NAAT. Consequently, if constraints related to the design of primers optimally suited for multiplex implementation were incorporated early in the discovery process, investigators could refine the down-selection of transcripts to satisfy these requirements.

## Genomic context of biomarkers

The success of cross-platform transfer can be influenced by the disparities in how high-throughput technologies employed for signature discovery and platforms used for clinical implementation capture the genomic context of differentially expressed loci. Gene expression microarrays (used before the advent of RNA-Seq) employ short probes (of usual length ∼25 base pairs) for quantification, whereas the region amplified in RT-qPCR is strictly defined by two primers. Thus, both techniques measure a subset of all the possible splicing variants for a particular gene, potentially missing biologically relevant transcripts. This also causes disparities between results of transcript quantification obtained on the two platforms: when the location of the microarray probe and RT-qPCR primers differ, the sets of splicing variants detected by microarray only, RT-qPCR only or both techniques might differ significantly.^25^

RNA-Seq is able to provide highly granular information on the exons whose expression is associated with a disease class. This information is very useful for designing assays where forward and reverse primers are located precisely within exons. Using this strategy, strong correlation in quantification between RNA-Seq and RT-qPCR has been demonstrated.^26^ This necessitates a DNAse pre-treatment of samples prior to the retro-transcription of mRNA into complementary DNA (cDNA), to prevent amplification of genomic DNA (which would lead to inaccurate measurement of transcript levels). However, it is challenging to remove all genomic DNA in a sample, and pre-treatment is currently incompatible with most point-of-care platforms.

Another common strategy to avoid the spurious amplification of genomic DNA is the ‘intron-spanning’ assay design, whereby for each amplicon the forward and reverse primers are positioned on two separate exons spanning at least one intron. However, even using such designs, discrepancies may remain between the sets of splicing variants detected by the two platforms, similar to what is seen with microarrays. We suggest that depending on the strategy used for avoiding amplification of genomic DNA, mRNA biomarker selection should be performed using measurements of exon or splice junction expression, in order to ensure biomarkers will measure the same transcripts in discovery and translation.^27^

## Future prospects

As we have shown, constraints related to the choice of a given implementation platform or chemistry are rarely taken into consideration in the initial steps of transcriptomics studies focusing on identifying the most informative gene signatures. This omission leads to both technical challenges in translation and to potential loss of classification performance in cross-platform transfer. We argue that focusing the discovery on specific genomic regions, rather than aggregate gene expression, and ensuring that these regions fulfil specific predefined criteria related to the chosen implementation platform and chemistry, could mitigate this loss in performance and improve the success rate of implementation efforts. We suggest that this set of constraints, i.e., the technical characteristics of the platform/chemistry, multiplexing strategy, and the genomic context of mRNA biomarkers, should be explicitly embedded in the down-selection algorithm used for sparse signature identification. Gene signatures identified through this process would be specific to a chosen implementation setting, but more likely to retain classification accuracy in clinical applications.

This simple paradigm shift would open exciting research avenues into the way integrating data analysis and molecular assay design can be used to facilitate faster and more reliable design of molecular biology experiments. What this approach implies is that among a large set of differentially expressed genes, only a subset of gene regions would be optimal candidates for platform transfer. However, identifying those ‘optimal’ gene regions would require bioinformatics and machine learning techniques to reduce the need for extensive experimentation (i.e., design of primers, assay optimization, determination of analytical characteristics for each gene region). Existing algorithms using thermodynamic information to predict primer binding, specificity and efficiency of NAATs, can be used to narrow down the list of potential NAAT primers.^28^ Recently, progress has been made to evaluate *in silico* large sets of PCR primers that would lower the risk of non-specific amplification in the highly-multiplexed setting.^29^ More complex strategies also include machine learning components that have been trained on large datasets of PCR experiments to predict outcomes of amplification reactions based on template and primer characteristics.^30^ In the future, we envision that the development of these techniques will make it possible to streamline the design of NAATs, and that these *in silico* predictions will become invaluable tools to accelerate the identification and translation of transcriptomics signatures for clinical application in a variety of diseases.

Recently, decreased costs and improvements in data analysis tools have made next-generation sequencing (NGS) platforms more accessible, including nanopore-based tools that facilitate fast and cost-effective long-read sequencing. These advancements open avenues for direct transcript abundance assessment in clinical samples, both during signature discovery and for bedside implementation. In theory, this could mitigate the decrease in diagnostic performance observed during the transition from discovery to implementation. However, while NGS-based clinical diagnostics may become prevalent in the future, extensive evaluation of their robustness and cost-effectiveness, particularly in gene expression analysis contexts, is still required. In conclusion, we anticipate that NAATs such as PCR will continue to play a valuable role in evaluating host response biomarkers across various clinical scenarios.

## Contributors

MK and JRM conceived and planned this Viewpoint. LK, GD’S, LM, and OV drafted the original version of the manuscript. LK and LM did the figure. All authors contributed to the intellectual development of this Viewpoint and provided critical feedback on the manuscript. The final version of this Viewpoint has been seen and approved by all authors.

## Declaration of interests

This article is the subject of a patent application filed by Imperial College London, Patent Application Publication No. WO2024023491A1, “A method to optimise transcriptomic signatures”, where JRM, DHC, HRJ, LM and MK are named inventors. All other declare no competing interests.
